# Health-Related Quality of Life in Children With Earlier Surgical Repair for Right Ventricular Outflow Tract Anomalies and the Agreement Between Children and Their Parents

**DOI:** 10.3389/fcvm.2020.00066

**Published:** 2020-04-28

**Authors:** Birgitta Svensson, Ewa Idvall, Fredrik Nilsson, Petru Liuba

**Affiliations:** ^1^Department of Cardiology, Pediatric Heart Centre, Skåne University Hospital and Lund University, Lund, Sweden; ^2^Department of Care and Science, Faculty of Health and Society, Malmö University, Malmö, Sweden; ^3^Clinical Studies Sweden-Forum South, Skåne University Hospital, Lund, Sweden

**Keywords:** complex right ventricular outflow tract anomalies, HRQoL, children, agreement, PedsQL questionnaire

## Abstract

**Background:** Children diagnosed with right ventricle outflow tract (RVOT) anomalies require surgical repair early in life, reoperations and lifelong follow-up. The aim is to comprehensively describe their health related quality of life (HRQoL) and to assess the agreement in this regard between children and parents.

**Methods and Results:** Child- and parent-reported HRQoL was assessed in 97 children aged 8–18 years using three different HRQoL questionnaires. The mean age was 12.9 ± 3 years. The mean total score for the child report was lower in the PedsQL Cardiac Module than in the PedsQL 4.0 and DISABKIDS (*p* ≤ 0.001). The mean score for each domain in PedsQL Cardiac Module ranged between 67 (cognitive function) and 79 (physical appearance), and between 72 (school function) and 82 (physical and social function) in PedsQL 4.0. Nearly half of the children reported problems with shortness of breath during physical activity. In the PedsQL Cardiac Module the child-parent agreement was strong for 13 of 22 items.

**Conclusion:** HRQoL problems as perceived by children with RVOT anomalies are best identified with the PedsQL Cardiac Module and relate mostly to cognitive and physical functioning. The agreement findings suggest the need to take into account both child- and parent reports in the assessment of HRQoL.

## Introduction

### Children With Right Ventricular Outflow Tract Anomalies

Children with RVOT anomalies include a heterogeneous group of congenital heart defects with need for early treatment and a multifaceted clinical assessment during follow-up. The surgical repair is usually performed during the first months of life and many of the children will eventually need one or more reoperations before adult age, often due to a leaking pulmonary valve or re-stenosis of the reconstructed outflow tract (1). Once a conduit is implanted, later re-interventions are necessary since conduits undergo degenerative changes and secondary worsening of the valve function. Delayed pulmonary valve replacement will result in adverse right ventricle remodeling with subsequently increased risk for cardiac events in later life, such as ventricular arrhythmias, heart failure and death. Optimizing time for re-intervention is therefore essential in order to avoid such complications (1). The relatively vague clinical symptoms in these children make it difficult to integrate the children's clinical status in the decision-making in terms of re-intervention and follow up. It has recently been suggested that health-related quality of life (HRQoL) can be useful when it comes to individualizing treatment decision making in children with congenital heart disease (CHD) (2). Furthermore assessment of HRQoL in pediatric patients can facilitate communication between children and health care professionals and hidden morbidities can be identified (3).

### Heath-Related Quality of Life (HRQoL)

HRQoL has been defined as “the influence of a specific illness, medical therapy, or health services policy on the ability of patients to both function in and derive personal satisfaction from various physical, psychological, and social life contexts” (2). During the past years, it has been proposed that HRQoL measurement could aid in medical care and improve communication among children, parents and healthcare professionals (4). Previous studies indicated that children with congenital heart disease (CHD) may have lower HRQoL than healthy children (5) and that children with severe heart disease have lower HRQoL than children with mild or moderate heart disease (6). Cardiopulmonary bypass and reoperations are risk factors for impaired HRQoL (7). In children with CHD we have previously reported a negative correlation between the number of surgeries and HRQoL total score using DISABKIDS chronic generic measure-short version (8). Children with tetralogy of Fallot, a common type of RVOT anomaly, need at last one surgery during their lifetime. Previous study observed comparable HRQoL between children with tetralogy of Fallot and healthy children (9). However, other studies have indicated a psychosocial impact of CHD even after successful repair and a satisfactory clinical status for children with tetralogy of Fallot (10) and worse psychosocial health than for healthy controls (11). Decreased HRQoL has also been reported in children with truncus arteriosus, another type of RVOT anomaly (12).

### Child- and Parent-Reports in HRQoL Measurement

Previously the assessment of HRQoL mainly relied upon parents' proxy reports, due to the belief that children lack the cognitive and linguistic skills to self-report HRQoL (13). Children from five years of age seem capable of reliably self-reporting HRQoL with an age-appropriate questionnaire (14). Such questionnaires provide the possibility of considering child-report as the standard in the assessment of HRQoL in the pediatric population. The parent-report may provide another perspective on the child's HRQoL (13). The level of agreement between children and parents has been an issue for discussion since parallel measurement (child and parent versions consist of same questions about the child's HRQoL) became available (15). Measurement of agreement is not only used as a validation for a new instrument but also to understand the difference between child-and parent- report (16). Traditionally, family-centered care (FCC) approach has been the gold standard in pediatric health care setting. Nowadays more child-centered care (CCC) approach by taking into account the child's perspective is recommended (17).

Previous research indicated that conduit operation at higher age is a risk factor for late mortality (18), and that higher age at first conduit operation is associated with a reduced risk of reintervention. These findings illustrate the importance of further research concerning optimal timing for pulmonary valve replacement. The clinical status is generally important in establishing the need and timing for reoperation. Since symptomatology in these patients is often vague, HRQoL assessment might provide an important complement in the decision making for pulmonary valve replacement. The aims of the present study were to comprehensively describe the self-reported HRQoL in children with right ventricular outflow tract anomalies, to evaluate which questionnaire best identifies HRQoL problems and to assess the agreement in this regard between children and their parents. In this study, an extended assessment with three earlier validated questionnaires was used to measure HRQoL using both child and parent-report.

## Materials and Methods

We conducted a cross-sectional study using a descriptive design with three questionnaires, two generic: the Pediatric quality of life Inventory (PedsQL 4.0), DISABKIDS chronic generic measure-short version and a disease-specific questionnaire for children with cardiac diseases: the PedsQL Cardiac Module. The DISABKIDS chronic generic measure-short version has been used as a patient related outcome in the Swedish national registry for congenital heart disease since the beginning of 2009 (19). PedsQL Cardiac Module has been available in the Swedish national registry for congenital heart disease since 2017.

### Study Population

The sample (*n* = 198) was obtained from the Swedish national registry for congenital heart disease (19) (*n* = 172) and from the surgical list (*n* = 26). The search was performed in September 2015 and children between 8 and 18 years old (born between 1997 and 2007) with various diagnoses concerning right ventricular outflow tract anomalies (Table 1) were included. Exclusion criteria were Mb Down, Trisomy18, chromosome disorder for chromosomes 4 and 13, Wolf Hirschhorn syndrome, heart surgery/intervention within three months, single ventricle and moving abroad after surgery.

**Table 1:** Surgical characteristics and diagnoses in the study population.

	**8–12 years *N* (%)**	**13–18 years *N* (%)**	**Total sample *N* (%)**
Number (N) of patients	47 (48)	50 (52)	97
^*^N of catheterizations	18 (38)	19 (38)	37 (38)
1	10 (56)	11 (58)	21 (57)
2	3 (17)	1 (5)	4 (11)
≥3	5 (27)	7 (37)	12 (32)
**N of cardiac surgery**			
1	19 (40)	18 (36)	37 (38)
2	14 (30)	22 (44)	36 (37)
≥3	14 (30)	10 (20)	24 (25)
Surgery with conduit	17 (36)	22 (44)	39 (40)
Without conduit	30 (64)	28 (56)	58 (60)
**Age at first surgery**			
<1 month	12 (26)	7 (14)	19 (20)
1–3 months	9 (19)	7 (14)	16 (17)
3 months–1 year	23 (49)	26 (52)	49 (51)
> 1 year	3 (6)	10 (20)	13 (13)
**Age at last surgery**		%50	
<1 year	25 (53)	17 (34)	42 (43)
1-7 years	15 (32)	15 (30)	30 (31)
8-17 years	7 (15)	18 (36)	25 (26)
**Cardiac defects**			
Tetralogy of Fallot	31 (66)	35 (70)	66 (68)
Fallot with PA	0	3 (6)	3 (3)
DORV (Fallot)	5 (11)	1 (2)	6 (6)
Acyanotic Fallot	0	2 (4)	2 (2)
PAVSD+MAPCA	8 (17)	1 (2)	9 (9)
TRUNCUS Arteriosus	0	1 (2)	1 (1)
DORV	3 (6)	5 (10)	8 (8)
PAVSD	0	2 (4)	2 (2)

* *Diagnostic catheterization included; PA: pulmonary atresia, DORV: double outlet right ventricle, VSD: ventricular septal defect, MAPCA: major aorta-pulmonary collateral arteries*.

### Data Collection

The three questionnaires, PedsQL 4.0, PedsQL Cardiac Module, and DISABKIDS chronic generic measure-short version child- and parent-report, together with an age-appropriate information letter, compliance and non-compliance form and health form were sent out by post. The information letter included detailed instruction about how to complete the PedsQL and how to administer the PedsQL to the child. The children and their parents completed the questionnaires at home and returned them in the enclosed envelope. After some weeks if no response had been obtained the first author telephoned the families following the instruction in the information letter. One reminder letter was sent out.

### PedsQL 4.0 and PedsQL Cardiac Module

PedsQL questionnaires include parallel child- and parent-report. PedsQL 4.0 consists of 23 items in four domains: physical functioning (eight items), emotional functioning (five items), social functioning (five items), and school functioning (five items). A physical health summary score is the same as the domain of physical functioning. To generate a psychosocial health summary score the mean is computed as the sum of the items divided by the number of items in the domains emotional, social, and school functioning (20). The PedsQL Cardiac Module is a disease-specific module and has 27 items in six domains: heart problem (seven items), treatment (five items), perceived physical appearance (three items), treatment anxiety (four items), cognitive problems (five items), and communication (three items) (21).

Both the PedsQL 4.0 and PedsQL Cardiac Modules were developed through focus groups and cognitive interviews and have been tested for reliability and validity (6, 20–22). Child- and parent-report used in this study includes the ages of 8–12 and 13–18 years. The difference between the various age scales is developmentally appropriate language (21). The instruction consists of how much of a problem each item has been during the last month. A five-point response scale is used (0 = never a problem; 1 = almost never a problem; 2 = sometimes a problem; 3 = often a problem, 4 = almost always a problem). The items were reverse-scored and linearly transformed to a 0 to 100 scale (0 = 100, 1 = 75, 2 = 50, 3 = 25, 4 = 0), so higher score indicated better HRQoL (21).

### DISABKIDS Chronic Generic Measure-Short Version

The DISABKIDS chronic generic measure-short version consists of child- and parent-report questionnaires earlier validated for use in children from 8 to 17 years of age with chronic disease (23) and was developed by “The DISABKIDS group Europe”, a cooperation project between Germany, Sweden, France, the Netherlands, Austria, and the United Kingdom (24). The questionnaire includes 12 items−10 items excluding medications—assessing mental (four items), social (four items), and physical (four/two items) impact on the health condition. Each question considers the last 4-week period, and the response for each item is graded using a 5-point scale, indicating frequency of behaviors or feelings as 1 = never, 2 = seldom, 3 = quite often, 4 = very often, and 5 = always. A higher score (maximum 100) indicates a better HRQoL (24).

### Statistical Analysis

For ease of comparisons with other previously published data in the field, our data are presented as means and standard deviation. Moreover, the central limit theorem ensures that the convergence of the distribution of the mean is rather quick for non-pathological distributions. Child and parent agreement was assessed by comparison of score means and by correlation. This was performed at three score levels: total-, domain and item level. A strong agreement between children and parent was defined as no significant difference in mean score combined with large correlation. The Wilcoxon test was used for comparison of means and Spearman correlation was used for correlation. For effect size (25): small *r* = 0.10–0.29, medium *r* = 0.30–0.49, and large *r* = 0.50–1.0. *p*-values less than 0.05 were considered significant (^*^ < 0.05, ^**^ < 0.01, ^***^ < 0.001). Statistical analyses were performed using R (26) while simple statistics were calculated using SPSS version 23.0 (SPSS IBM, New York, United States of America). Dropout analysis was performed using Fisher's Exact test concerning age, gender, diagnoses (TOF). In order to compare the total score in child-reported PedsQL 4.0, PedsQL Cardiac Module and DISABKIDS chronic generic measure-short version a linear mixed model (nlme 3.1–137) was used to account for repeated measures (27). *Post-hoc* comparisons were made using the package multcomp 1.4–8 (28) adjusting for multiplicity. The results were corroborated using a linear mixed model. Internal consistency for each scale and for the whole questionnaires was assessed using the Cronbachs alfa. In PedsQL 4.0 and PedsQL Cardiac Module the domain score was computed as the sum of the items divided by the number of items answered (accounts for missing data). If more than 50% were missing the domain sum was not computed (20). In our study, the frequencies of “3 = often a problem” and “4 = almost always a problem” were merged and ranked to identify the items children reported most problems with, in the PedsQL 4.0 and PedsQL Cardiac Module.

The total score from the DISABKIDS chronic generic measure-short version was transformed from a raw score to a range of 0–100 (24). Thus, a “standard raw score” = ((point −12)/48) X 100 (if 12 questions were answered)) or = ((point −10)/40) X 100 (if 10 questions were answered)). The responses were transformed so 5 (always) indicated better HRQoL and 1 (never) indicated lower HRQoL in items 3–8 and 11–12. In our study, the answers “never” or “seldom” and “always” or “often” were merged depending on the value course for the items, to identify the items that the children rated lowest HRQoL in DISABKIDS chronic generic measure-short version.

## Results

The response rate was 50% (*n* = 97). The mean age of this cohort was 12.9 ± 3 years; 59 (61%) were boys and 47 (48 %) were children from 8 to 12 years old. Parent responders were 64% mothers, 17% fathers, 12% both, and 7 % unknown. The diagnoses and surgical data are shown in Table 1. The mean time between the last surgery and responded questionnaire was 8.9 ± 4.9 years. Fifteen (17%) children were on pharmacological therapy due to their heart condition. Two child-reports and two parent-reports were excluded because the parents informed that they had completed the child-reports. The PedsQL 4.0 was completed in 90 child- and parent-reports (93%). The PedsQL Cardiac Module was completed in 93 child-reports (96 %) except for the domain treatment II (*n* = 14, 16 %) and in 90 parent-reports (93 %) except for the domain treatment II (*n* = 15, 17 %). The domain treatment II in PedsQL Cardiac Module was excluded in the results, because of few children on medication. The DISABKIDS chronic generic measure-short version was completed in 91 child-and parent-reports (94%). No significant differences (*p* ≥ 0.3) were found in dropout analysis (Table 2) in terms of gender, age groups and diagnosis (Tetralogy of Fallot). Cronbachs α for both PedsQL 4.0 and PedsQL Cardiac Module at domain level and for the whole questionnaires varied between 0.70 and 0.95. Cronbachs α for the DISABKIDS chronic generic measure-short version was 0.9.

**Table 2:** Drop out analysis.

	**N**	**Dropout**	***p*-value**
Girls/boys	38/59	36/63	>0.3
8-12 år/13-18 år	47/50	46/53	>0.3
Fallot/others	66/31	64/35	>0.3

*Frequencies and Fisher's Exact test*.

### Child-Reported HRQoL

The mean total score (highest HRQoL; 100) for the child-report was significant lower in the PedsQL Cardiac Module (Table 3) than in PedsQL 4.0 (*p* ≤ 0.012: CI=1.21-6.50) and DISABKIDS chronic generic measure-short version (*p* ≤ 0.001: CI=4.17-9.42). The mean child-report score for each domain in PedsQL Cardiac Module ranged between 67 (cognitive function) and 79 (physical appearance), and between 72 (school function) and 82 (physical and social function) in PedsQL 4.0 (Table 3). Up to 20% of the children reported problems regarding limited physical capacity and cognitive function in the PedsQL 4.0. Nearly half of the children reported problems with breathlessness when participating in sports, a quarter reported cognitive and communication problems and 14% of the children reported problems in the PedsQL Cardiac Module regarding other people seeing their scar (Table 4). A total of 10% of children reported in the DISABKIDS chronic generic measure-short version that their heart disease caused them different kinds of troubles or hard feelings (Table 5).

**Table 3:** Mean score and standard deviations (SD) for the child and parent reports in PedsQL cardiac module, PedsQL 4.0, and DCGM-12.

**Total sample**	**Self**		**Parent**			
	**Mean**	**SD**	**Mean**	**SD**	***p***	***r***
**PedsQL 4.0**	*N* = 90		*N* = 89			*N* = 85
Physical functioning	82	19	79	22	0.067	0.84^***^
Emotional functioning	76	22	72	23	0.033^*^	0.82^***^
Social functioning	82	20	78	24	0.055	0.78^***^
School functioning	72	22	70	23	>0.3	0.80^***^
Psychosocial^a^	77	20	73	21	0.049^*^	0.85^***^
Total score	78	18	75	21	0.040^*^	0.86^***^
**PedsQL cardiac module**	*N* = 93		*N* = 90			*N* = 87–88
Heart problems	76	18	77	20	>0.3	0.80^***^
Physical appearance	79	24	80	23	>0.3	0.49^***^
Treatment anxiety	74	29	76	29	>0.3	0.65^***^
Cognitive problems	67	26	62	29	0.065	0.83^***^
Communication	74	27	71	31	>0.3	0.61^***^
Total score	74	19	74	20	>0.3	0.73^***^
**DCGM-12**	*N* = 91		*N* = 91			*N* = 86
Total score	81	21	81	23	>0.3	0.78^***^
Median (10–90)	88	55-100	90	48-100		

*a: aggregated emotional+social+school. Column p represents p-value from Wilcoxon's test for mean rank comparison between child and parent report*.

* p < 0.05 and

*** *< 0.001. Column r: Spearman correlation between child and parent report*.

**Table 4:** Items for specific concerns by child report in PedsQL cardiac module and PedsQL 4.0.

**PedsQL 4.0 *N* = 90**	**Child self report *N* (%)**
**Almost always or often problem with**	
It is hard for me to run	17 (19)
I have low energy	16 (18)
I worry what will happen to me	15 (17)
It is hard to pay attention in class	15 (17)
I forget things	18 (20)
I have trouble keeping up with my schoolwork	14 (16)
**PedsQL cardiac module (*****N*** **=** **90–93)**	
I get out of breath when I do sports activity or exercise	39 (43)
I have to rest more than my friends	20 (22)
I have troubling solving math problems	23 (25)
It is hard for me to remember what I read	18 (19)
It is hard for me to pay attention to things	17 (18)
It is hard for me to explain my heart problem to other people	16 (17)

**Table 5:** Items for specific concerns by child report in DCGM-12.

**DCGM-12 *N* = 90**	**Child self report *N* (%)**
**Never or seldom**	
Do you feel like everyone else even though you have your condition?	9 (10)
**Always or often**	
Is your life ruled by your condition	9 (10)
Does your condition bother you when you play or do other things?	10 (11)
Are you unhappy because of your condition	11 (12)
Do you feel different from other children/adolescents?	10 (11)

### Agreement Between Child- and Parent-Report for Each Questionnaire

**Figure 1:**
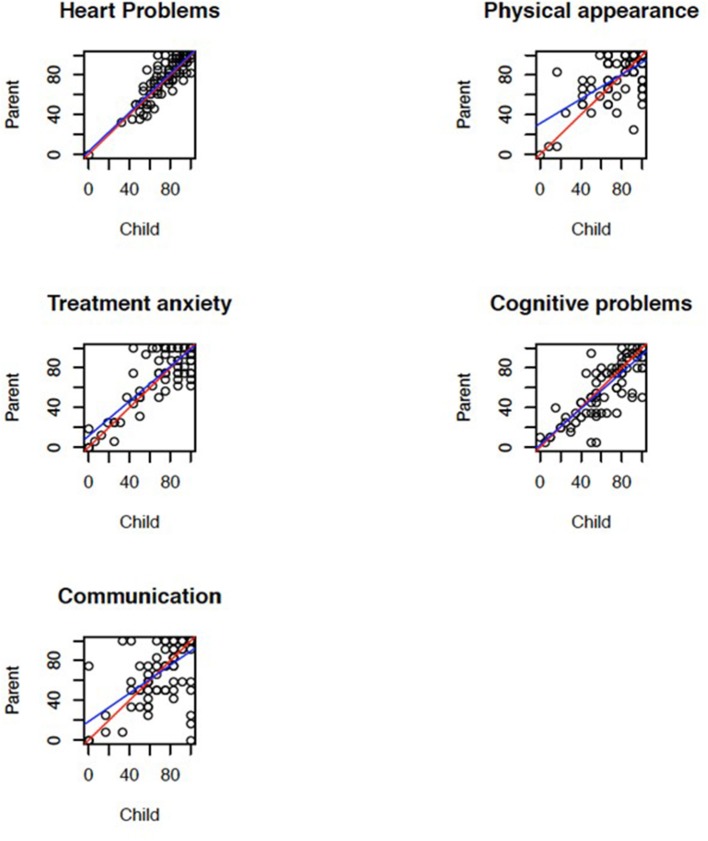
Scatterplot with each point corresponding to a child-parent pair to show the correlation between children and parents for the domains of the PedsQL cardiac module. The red line is perfect agreement and the blue line is the robust least-squares fit.

In the PedsQL 4.0 the agreement was strong between children and parents for the domains physical-, social- and school function and the correlation was large in all domains (Table 3). Children rated significantly higher HRQoL than their parents for the domain emotional function and for total score. In the PedsQL Cardiac Module the agreement was strong between children and parents for all domains except physical appearance where the correlation was medium (Figure 1). In the DISABKIDS chronic generic measure-short version the agreement was strong for total score.

### Agreement Between Child- and Parent-Report for Different Age Groups at Domain and Item Levels in the PedsQL Cardiac Module

#### Children 8-12 Years of Age

In this subgroup of children the agreement was strong between children and parents for all domains except for perceived physical appearance (Table 6). At item level the agreement was strong for 13 (of 22) items. The correlation was medium between children and parents (*r* = 0.3–0.49) for two (of seven) items in domains heart problems, for two (of three) in perceived physical appearance and for two (of three) in communication. For all other items the correlation was large (*r* ≥ 0.5). Among these children the lowest HRQoL was rated for the item ‘I get out of breath when I do sports activity or exercise' and children rated this significantly lower than their parents. Children rated significantly higher HRQoL than their parents for one item and lower for two items of a total of 22 items (Table 6).

**Table 6:** Agreement between children and parents at item level for the two age groups in PedsQL cardiac module.

**PedsQL cardiac module**	**Self 8–12**		**Parents 8–12**				**Self 13–18**		**Parent 13–18**			
**How much of a problem has this been for you?**	**Mean *N* = 41-44**	**SD**	**Mean *N* = 43-45**	**SD**	***P***	***r***	**Mean *N* = 48–49**	**SD**	**Mean *N* = 45–46**	**SD**	***p***	***r***
Heart problems	75	17	75	21	>0.3	0.813^***^	77	19	78	20	0.083	0.787^***^
1. I get out of breath when I do sports activity or exercise	43	26	54	32	0.048^*^	0.760^***^	50	34	52	34	>0.3	0.819^***^
2. My chest hurts or feel tight when I do sports activity or exercise	77	22	82	25	0.15	0.493^**^	84	23	86	22	>0.3	0.620^***^
3. I catch colds easily	76	27	75	30	>0.3	0.764^***^	70	29	78	24	0.002^**^	0.605^***^
4. I feel my heart beating fast	65	32	66	33	>0.3	0.773^***^	72	30	77	27	0.116	0.734^***^
5. My lips turn blue when I run	94	15	86	23	0.005^**^	0.537^***^	92	21	93	19	>0.3	0.510^***^
6. I wake up at night with troubling breathing	99	5	95	11	0.063	0.486^**^	97	16	95	17	0.25	0.655^***^
7. I have to rest more than my friends	66	35	67	32	>0.3	0.683^***^	71	31	68	34	>0.3	0.627^***^
Perceived physical appearance	83	20	82	22	> 0.3	0.374^*^	76	28	79	24	>0.3	0.580^***^
1. I feel I am not good looking	80	32	84	25	>0.3	0.364^*^	71	32	77	28	0.129	0.600^***^
2. I don‘t like other people to see my scars	78	32	78	31	>0.3	0.497^***^	78	34	81	27	>0.3	0.704^***^
3. I am embarrassed when others see my body	90	20	83	28	00.051	0.553^***^	78	33	79	27	>0.3	0.471^***^
Treatment anxiety	73	31	75	32	0.16	0.719^***^	76	28	77	27	>0.3	0.526^***^
1. I get scared when I am waiting to see the doctor	70	32	75	34	0.044^*^	0.827^***^	77	28	79	26	>0.3	0.666^***^
2. I get scared when I have to go to the doctor	75	35	77	34	0.183	0.782^***^	77	31	80	30	>0.3	0.499^***^
3. I get scared when I have to go the hospital	72	32	73	32	>0.3	0.808^***^	74	32	76	31	>0.3	0.452^**^
4. I get scared when I have to have medical treatments	73	31	74	34	>0.3	0.630^***^	76	33	76	32	>0.3	0.494^***^
Cognitive problems	66	26	64	27	> 0.3	0.848^***^	67	26	61	31	0.074	0.831^***^
1. It is hard for me to figure out what to do when something bothers me	63	32	66	30	0.156	0.691^***^	73	28	67	31	0.176	0.704^***^
2. I have troubling solving math problems	66	33	67	30	>0.3	0.841^***^	59	36	51	38	0.019^*^	0.867^***^
3. I have trouble writing school papers or reports.	70	30	62	31	0.051	0.672^***^	70	31	63	32	0.062	0.746^***^
4. It is hard for me to pay attention to things	64	32	59	35	>0.3	0.707^***^	68	31	59	34	0.040^*^	0.625^***^
5. It is hard for me to remember what I read	69	32	65	30	0.204	0.699^***^	65	35	64	36	>0.3	0.699^***^
Communication	75	24	76	30	> 0.3	0.513^***^	74	20	66	32	0.008^**^	0.740^***^
1. It is hard for me to tell the doctors and nurses how I feel	74	29	77	31	>0.3	0.577^***^	78	31	69	35	0.015^*^	0.642^***^
2. It is hard for me to ask the doctors and nurses questions	78	31	78	32	>0.3	0.458^**^	76	33	65	36	0.004^**^	0.683^***^
3. It is hard for me to explain my heart problem to other people	72	33	72	35	>0.3	0.385^*^	68	33	63	34	0.218	0.714^***^

*Agreement assessed by Wilcoxon signed ranks test for comparison of means and Spearman correlation*.

* *p < 0.05*,

** *< 0.01*,

*** *< 0.001*.

#### Children 13-18 Years of Age

In this subgroup of children the agreement was strong between children and parents for all domains except communication (Table 6). The agreement was strong for 13 (of 22) items. The correlation was medium for three items in the domain treatment anxiety and for the item “embarrassed when other sees my body.” For all other items the correlation was large. Among these children the lowest HRQoL was rated for item “I get out of breath when I do sports activity or exercise.” The parents rated lowest for the item “troubles solving math problems.” Children rated their HRQoL significantly higher than their parents for four items, all in the domains cognitive problems and communication, and lower for one item in the domain heart problems (Table 6).

## Discussion

Given the complexity of the studied cohort, we used three instruments to assess HRQoL. DISABKIDS chronic generic measure-short version was the first HRQoL instrument used in the Swedish national registry for congenital heart disease (19) in 2009 and later replaced by PedsQL Cardiac Module. PedsQL 4.0 has been used in previous studies (21, 29, 30). DISABKIDS chronic generic measure-short version has fewer questions, requiring thus shorter time to complete this questionnaire, but provides less detailed information regarding the physical and psychosocial functioning compared with the PedsQL questionnaires. The higher total score in the DISABKIDS chronic generic measure-short version and the lower percentage of children who reported difficulties could be related to these drawbacks, particularly in a cohort of children whose symptoms are less obvious. Our findings indicate that problems perceived by children were more easily detected with the disease-specific questionnaire PedsQL Cardiac Module, a result that supports the superiority of disease-specific questionnaires in assessing HRQoL in children with complex CHD (31). Furthermore, PedsQL questionnaires have the advantage of assessing HRQoL at both domain and item level thus enabling the opportunity to obtain more detailed and multifaceted information regarding HRQoL. Another difference between DISABKIDS chronic generic measure-short version and PedsQL is that PedsQL questionnaires are problem-based, being possibly more useful in the clinical follow up.

In a previous Swedish study on children with various types of CHD (32) a strong association was found between children and their parents' ratings of cognitive problems based on the PedsQL Cardiac Module and their intelligence score based on the Wechsler intelligence scales. According to Buratti the cognitive domain in PedsQL Cardiac Module could be used as a screening tool, with a cut off score of 80, to identify children needing standardized cognitive testing. In our study ~44 % (*n* = 93) of children had a PedsQL score for the cognitive domain below 80. Importantly the lowest score was in the domains school functioning (PedsQL 4.0) and cognitive problems (PedsQL Cardiac Module).

Sixteen of 93 children in our study reported difficulties in explaining their heart problem to other people (PedsQL Cardiac Module). Children with CHD need strategies to communicate about their heart disease (33). A possible reason for these difficulties may be that children do not have the knowledge or the language to talk about their heart disease. Improved knowledge in this regard has been showed to have a positive influence on the HRQoL for adolescents with CHD (34). Healthcare professionals may have a central role by providing age-appropriate information about the heart disease.

Physical capacity is an important determinant of HRQoL. One earlier PedsQL study found a positive correlation between the ability to exercise and the HRQoL among children with Tetralogy of Fallot (9). In our study, the mean scores for the domains of physical functioning and heart problems were 82 and 79, respectively. Item analysis in PedsQL Cardiac Module showed that nearly 50% of the children reported problems due to shortness of breath during physical activity indicating lower physical functioning for these children. This finding illustrates the advantage of item analysis, which may reveal further information not apparent at domain level.

The agreement in our study was assessed on both an individual level (correlation) and a group level (comparison of means), which is recommended to obtain a full assessment of agreement (35). Therefore, in our study a strong agreement was defined as a large correlation with no significant difference in mean score. The importance of using this approach can be explained as follows: although parents and children on average agree, the individual pair is not in complete agreement. Thus one contains information about the other. The picture is therefore complete only when both views are integrated. This result supports the previous findings (21, 29, 30) namely a need for both child- and parent-reports for children from 8 to 18 years of age. With the advent of the CCC approach in pediatric health care, the child's self report has become central whereas the parent report is considered n important complement. A key-element in the CCC approach is the right of especially older children to be informed and understand the diagnostic and therapeutic decision making. In a recent study (36), children with various chronic diseases reportedly felt encouraged, got insight about their health and improved their understanding of how the disease affected their life when they received feedback from health care professionals about the results of HRQoL assessment. Children with earlier corrected RVOT anomalies could substantially benefit from this since their clinical symptoms are often vague, being difficult to perceive and explain to health care providers. Applying this concept in their follow up could thus improve the understanding on their well-being and intuitively, add important information in the decision making for reoperation especially in those cases with regurgitant pulmonary valve.

### Study Limitations

(1) Although the response rate was only 50%, the dropout analysis showed no differences between the groups in terms of age, gender and diagnoses. (2) The cross-sectional study design may preclude assessment of changes in HRQoL over time. Therefore future prospective studies are important to assess these changes in children with these diagnoses. In order to mitigate the influence of time on the measured indices, we have included only patients with surgical interventions performed at least three months prior to the questionnaire. The questionnaires used in our study have excellent reproducibility over time in patients with chronic disease (21). (3) Another potential limitation is that the questionnaires were completed at home, possibly leading to biases due to potential parental support. However, as emphasized by Upton et al. (13) the place of completion (home vs. clinic) does not appear to influence the results in this regard. (4) Further studies including control population are needed in order to confirm that the difference in agreement between parents and children are specific to patients with RVOT anomalies.

## Conclusion

Our findings indicate that HRQoL perceived by children with right ventricular outflow tract anomalies is better assessed in the disease-specific questionnaire PedsQL Cardiac Module, suggesting that the PedsQL Cardiac Module is useful in the clinical follow up of these children. Problems concerning cognitive function should be considered by healthcare professionals as both children and parent reported the lowest HRQoL in this domain. The findings regarding the agreement between children with right ventricular outflow tract anomalies and their parents suggests the need to take into account both child- and parent- reports in the assessment of HRQoL. Further longitudinal research including interviews with both children and parents is important as this approach provides deeper understanding of HRQoL over time.

## Data Availability Statement

All datasets generated for this study are included in the article/supplementary material.

## Ethics Statement

This study was conducted in accordance with the ethical standard and the Helsinki Declaration and its later amendments. The study was approved by the Ethics Committee for Human Research at the Lund University (#2014/66). Informed consent was obtained from all study participants.

## Author Contributions

BS contributed to study design and statistical analyses, performed the study work and drafted the revised the manuscript. EI contributed to study design, review in the field of quality of life, revised the manuscript. FN was responsible for statistical analyses and reviewed the statistical part of the manuscript. PL contributed to study design and manuscript review.

## Conflict of Interest

The authors declare that the research was conducted in the absence of any commercial or financial relationships that could be construed as a potential conflict of interest.
